# Immunohistochemical localization of tumor necrosis factor-α in gastrointestinal tissues of budgerigars naturally infected with *Macrorhabdus ornithogaster*

**DOI:** 10.1007/s11259-026-11330-0

**Published:** 2026-06-13

**Authors:** Damla Atay, Ozlem Ozmen

**Affiliations:** 1 Antalya Metropolitan Municipality Natural Life Park, Antalya, Türkiye; 2 Department of Pathology, Faculty of Veterinary Medicine, Burdur Mehmet Akif Ersoy University, Istiklal Yerleskesi, 15030 Burdur, Türkiye

**Keywords:** *Macrorhabdus ornithogaster*, Budgerigar, TNF-α, Immunohistochemistry, Proventriculus, Avian pathology

## Abstract

Macrorhabdosis is a contagious fungal gastrointestinal disease of birds, that predominantly affecs the proventriculus of budgerigars (*Melopsittacus undulatus*). Although the pathological features of *Macrorhabdus ornithogaster* infection have been extensively described, information regarding local inflammatory cytokine responses in naturally infected birds remains limited. In the present study, archival gastrointestinal tissues obtained from 25 budgerigars naturally infected with *M. ornithogaster* and 5 non-infected controls were evaluated histopathologically and immunohistochemically. Routine histopathology was performed to characterize tissue lesions, while immunohistochemistry was used to evaluate tissue expression of tumor necrosis factor-alpha (TNF-α). Histological lesions were predominantly localized to the proventriculus and consisted of mucosal erosion, ulceration, epithelial degeneration, and inflammatory cell infiltration associated with abundant fungal organisms. PAS and Gomori methenamine silver staining confirmed the presence and distribution of fungal elements within affected tissues. TNF-α immunoreactivity was detected mainly in epithelial and inflammatory cells within lesion affected areas, whereas control tissues exhibited minimal or no immunostaining. These findings demonstrate localized TNF-α expression in gastrointestinal tissues affected by *M. ornithogaster* infection and support the presence of an active TNF-α inflammatory response associated with macrorhabdosis. This study provides a descriptive baseline data for future quantitative and functional investigations evaluating cytokine-mediated mechanisms in avian fungal gastrointestinal disease.

## Introduction

*Macrorhabdus ornithogaster* is an anamorphic ascomycetous yeast belonging to the order *Saccharomycetales* that primarily colonizes the isthmus between the proventriculus and ventriculus of avian hosts (Gerlach 2001). The organism is most commonly detected in birds of the orders *Psittaciformes*,* Passeriformes*, and *Galliformes*, with budgerigars (*Melopsittacus undulatus*), small parrots (*Forpus* spp.), and canaries (*Serinus canaria*) being particularly susceptible (Phalen 2005; Martins et al., 2006; Hannafusa et al. 2007; Phalen 2014; Borrelli et al. 2015). Although infections are frequently subclinical, clinically affected birds may exhibit progressive weight loss, regurgitation, melena, and, in severe cases, sudden death (Dorrestein et al. 1980; Phalen 2005). Both acute and chronic disease forms have been described, with chronic macrorhabdosis often characterized by persistent digestive disturbances and malabsorption (Phalen et al., 2002; Phalen 2006; Snyder and Treuting 2014; Amer and Mekky 2020).

Pathological lesions associated with *M. ornithogaster* infection predominantly involve the proventriculus and, to a lesser extent, the ventriculus, where mucosal erosion, ulceration, and inflammatory changes are commonly observed. In contrast, intestinal involvement has been reported only sporadically (Kheirandish and Salehi 2011; Ozmen et al. 2013; Powers et al. 2019). Diagnosis is primarily based on microscopic identification of the organism in fecal samples or gastrointestinal tissues, where *M. ornithogaster* appears as elongated, rod-shaped structures that stain positively with periodic acid–Schiff (PAS), Gram, and Giemsa stains. Despite its clinical relevance, treatment options remain limited and variably effective, underscoring the importance of improving our understanding of disease pathogenesis.

Tumor necrosis factor-alpha (TNF-α) is a key proinflammatory cytokine produced mainly by monocytes and macrophages and plays a central role in host defense, immune regulation, and inflammatory responses. Through its effects on epithelial and immune cells, TNF-α contributes to both protective mechanisms against pathogens and tissue injury in chronic inflammatory conditions (Darnay and Aggarwal 1999; Idriss and Naismith 2000; Pobezinskaya and Liu 2012). While the role of TNF-α has been extensively studied in mammalian infectious and inflammatory diseases, data regarding its expression and localization in avian fungal infections remain scarce.

Considering the marked inflammatory alterations associated with macrorhabdosis, characterization of local cytokine expression may provide important insight into host–pathogen interactions and tissue injury mechanisms. In this context, the present study aims to describe the immunohistochemical expression of TNF-α in gastrointestinal tissues of budgerigars naturally infected with *M. ornithogaster*. By documenting the spatial distribution of TNF-α in relation to fungal colonization and associated lesions, this study seeks to contribute descriptive evidence toward understanding the inflammatory response in avian macrorhabdosis and to provide a basis for future quantitative and functional investigations.

## Materials and methods

### Cytological examination

Cytological examination of proventricular imprint smears is routinely performed in cases with suspected gastrointestinal disease or clinical suspicion of macrorhabdosis as part of the diagnostic workup in our laboratory. During necropsy, fresh proventricular and, when available, small intestinal tissues were gently pressed onto clean glass slides to obtain imprint smears. The smears were air-dried at room temperature, fixed in absolute methanol for 5 min, and stained with Giemsa using standard procedures. Slides were examined under a light microscope to support the identification of *M. ornithogaster.* Cytological findings were used solely as a supportive diagnostic tool and were not included in comparative or quantitative analyses.

### Tissue samples and histological procedures

Precise age and treatment histories were not available for all archived cases. Based on submission records, most birds were adult budgerigars presented with chronic weight loss, diarrhea, or gastrointestinal signs suggestive of macrorhabdosis. None of the birds had complete clinical follow-up information available. This study was conducted using archived formalin-fixed, paraffin-embedded (FFPE) gastrointestinal tissue samples obtained from the archives of the Department of Pathology. All materials originated from routine diagnostic submissions; therefore, no additional animal use was involved, and ethical approval was not required. The study was supported by the Scientific Research Projects Coordination Unit of Burdur Mehmet Akif Ersoy University (Project No. 0995-YL-24).

A total of 25 FFPE gastrointestinal tissue blocks from budgerigars diagnosed with macrorhabdosis were included. Samples were mainly derived from the proventriculus and ventriculus, while intestinal tissues were evaluated when available. Five budgerigars that died from causes unrelated to M. ornithogaster infection and tested negative for the organism were used as controls.

Tissue Sect.  (5 μm) were prepared using a rotary microtome (Leica RM2155, Leica Microsystems, Germany) and mounted on standard glass slides for routine histology and on poly-L-lysine–coated slides for immunohistochemistry. Following deparaffinization and rehydration, hematoxylin and eosin (H&E) staining was performed. Selected sections were additionally subjected to Periodic Acid-Schiff (PAS) staining (PAS Stain Kit for Fungi, Abcam, ab245886) and Gomori methenamine silver (GMS) staining (GMS Stain Kit, Abcam, ab287884) according to manufacturer protocols to confirm fungal elements. All slides were examined using a light microscope (Zeiss Axioscope 5), and representative images were captured.

### Immunohistochemical analysis for TNF-α

Immunohistochemical analysis for TNF-α was performed on infected and control gastrointestinal tissues using a rabbit polyclonal anti–TNF-α antibody (Novus Biologicals, NB600-587SS), reported to exhibit cross-reactivity with avian TNF-α, at a dilution of 1:200. Poly-L-lysine–coated sections were deparaffinized and rehydrated. Heat-induced antigen retrieval was performed in citrate buffer (pH 6.0) using a microwave oven at 700 W for two cycles of 5 min. Endogenous peroxidase activity was blocked by incubation in 3% hydrogen peroxide for 10 min at room temperature., and sections were incubated overnight at 4 °C with the primary antibody. Immunodetection was performed using an UltraVision Detection System Anti-Polyvalent HRP (TP-060-HL, Thermo Fisher Scientific, USA) with 3,3′-diaminobenzidine (DAB) as the chromogen. Sections were counterstained with Harris hematoxylin, dehydrated, cleared, and mounted. Negative controls were prepared by omission of the primary antibody, while tissues containing inflammatory cell populations served as internal positive controls. All stained sections were independently evaluated by experienced veterinary pathologists using light microscopy.

### In silico sequence analysis of TNF-α

To evaluate the suitability and potential cross-reactivity of the anti-TNF-α antibody used in immunohistochemical analyses, both in silico comparative sequence analysis and information provided by the manufacturer and previously published studies were considered. The amino acid sequence of tumor necrosis factor-α (TNF-α) from *Melopsittacus undulatus* (budgerigar; NCBI Gene ID: 117438142) was retrieved from the NCBI database. Orthologous TNF-α protein sequences from representative vertebrate species (*Gallus gallus*,* Homo sapiens*,* Mus musculus*, and *Rattus norvegicus*) were also obtained. The multiple sequence alignment demonstrated that TNF-α is highly conserved among vertebrates. The *Melopsittacus undulatus* TNF-α sequence showed the highest similarity with *Gallus gallus*, consistent with avian phylogenetic proximity. Conservation was particularly pronounced within the TNF-α homology domain, including residues involved in receptor-binding, which are known to represent key antigenic regions targeted by commercially available anti-TNF-α antibodies.

In addition, according to the manufacturer’s datasheet and previously published validation studies, the antibody has been reported to recognize conserved epitopes within the TNF-α protein across multiple vertebrate species. These independent sources further support the likelihood of cross-reactivity in avian tissues. Amino acid identity analysis corroborated these findings by quantifying the highest similarity between *Melopsittacus undulatus* and *Gallus gallus*, followed by mammalian species, thereby providing a robust quantitative framework for interpretation. Phylogenetic analysis supported these results, revealing a clear separation between avian and mammalian clades while confirming the close evolutionary relationship between *Melopsittacus undulatus* and *Gallus gallus*. This pattern is consistent with the expected evolutionary conservation of TNF-α among vertebrates (Marín 2020; Lu et al. 2022).

Importantly, immunohistochemical specificity was further validated using appropriate negative controls, in which omission of the primary antibody resulted in complete absence of staining. Collectively, in silico data, manufacturer-provided validation information, and previously published studies provide complementary evidence supporting the specificity and reliability of TNF-α immunodetection in budgerigar tissues.

## Results

**Fig. 1:**
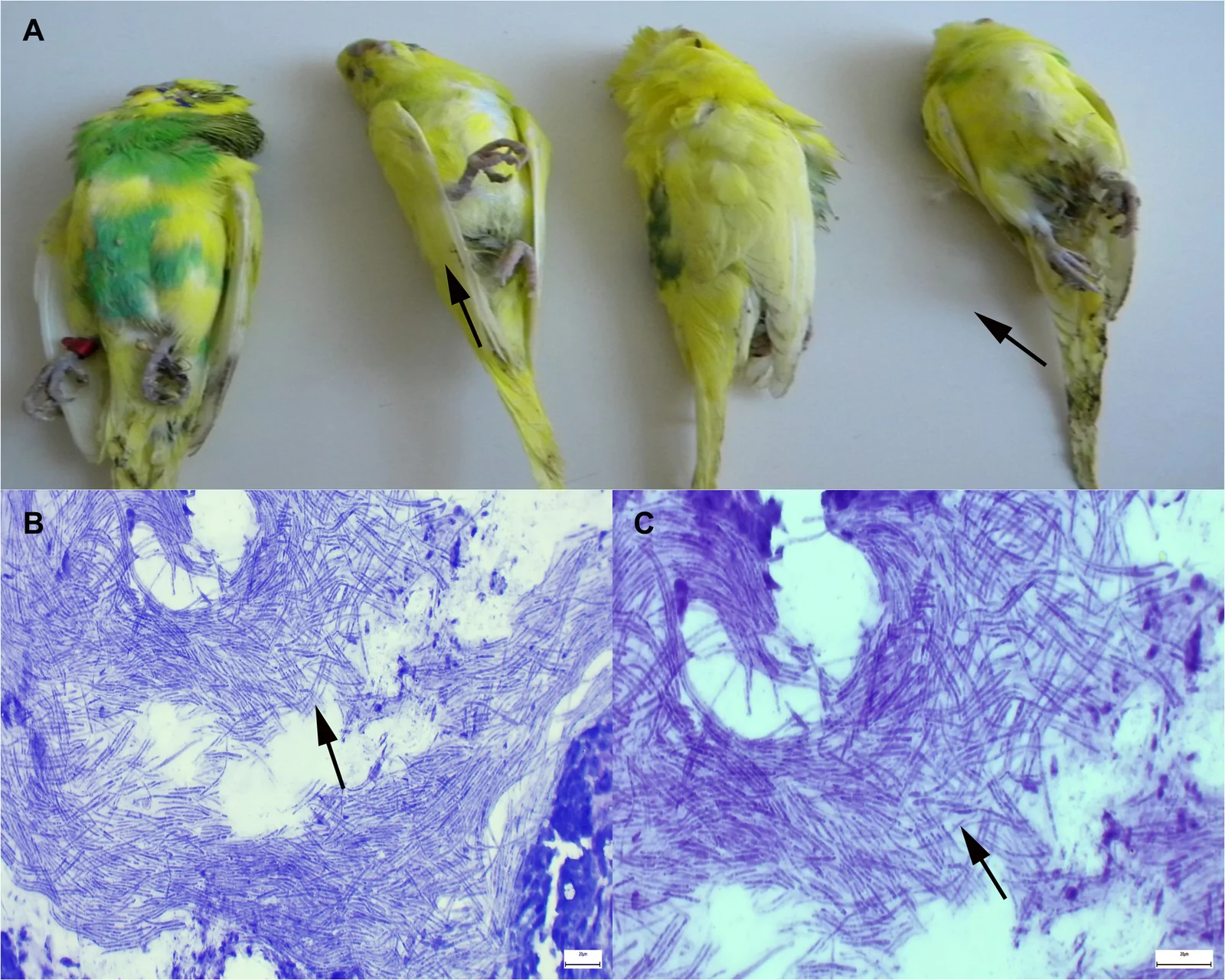
(**A**) Severe emaciation, prominence of the sternal keel, and diarrhea in budgerigars with macrorhabdosis. (**B**) Proventricular cytology of budgerigars that died from macrorhabdosis. A sample with a very high density of organisms (arrow), Giemsa, 50 μm, (**C**) Enlarged view of the organisms (arrow), Giemsa, 20 μm

Gross examination of budgerigars submitted for necropsy with a clinical suspicion of macrorhabdosis revealed consistent alterations in body condition and the upper gastrointestinal tract. Most birds appeared clinically cachectic, with poor body condition and variable prominence of the sternal keel (Fig. 1A). Diarrhea was observed in several birds and ranged from mildly watery droppings to profuse, greenish feces, occasionally containing undigested food material. No other consistent external gross abnormalities were noted. Examination of the upper gastrointestinal tract revealed macroscopic changes primarily affecting the proventriculus and, less frequently, the ventriculus. The proventricular wall appeared thickened in multiple cases and was often covered by excessive amounts of viscous mucus. Multifocal areas of mucosal erosion and ulceration were observed, occasionally accompanied by petechial to more extensive hemorrhages. In some birds, the koilin layer of the ventriculus appeared irregular, discolored, or partially detached. In these cases, hemorrhagic material and blood clots were present within the proventriculus–ventriculus lumen. Overall, the gross pathological findings were predominantly localized to the upper gastrointestinal tract, with the most pronounced changes observed in the proventriculus.

Cytological examination of proventricular imprint smears revealed the presence of elongated, rod-shaped organisms consistent with *M. ornithogaster*. Using Giemsa staining, organisms appeared as blue to purple filamentous structures, occurring singly or in clusters within the preparations. At higher magnification, the organisms exhibited a segmented appearance with transverse banding, characteristic of *M. ornithogaster*. No additional cytological abnormalities were consistently observed. Cytological findings were considered supportive of the histopathological diagnosis and were not evaluated in relation to lesion severity or immunohistochemical results (Fig. 1B and C).

**Fig. 2:**
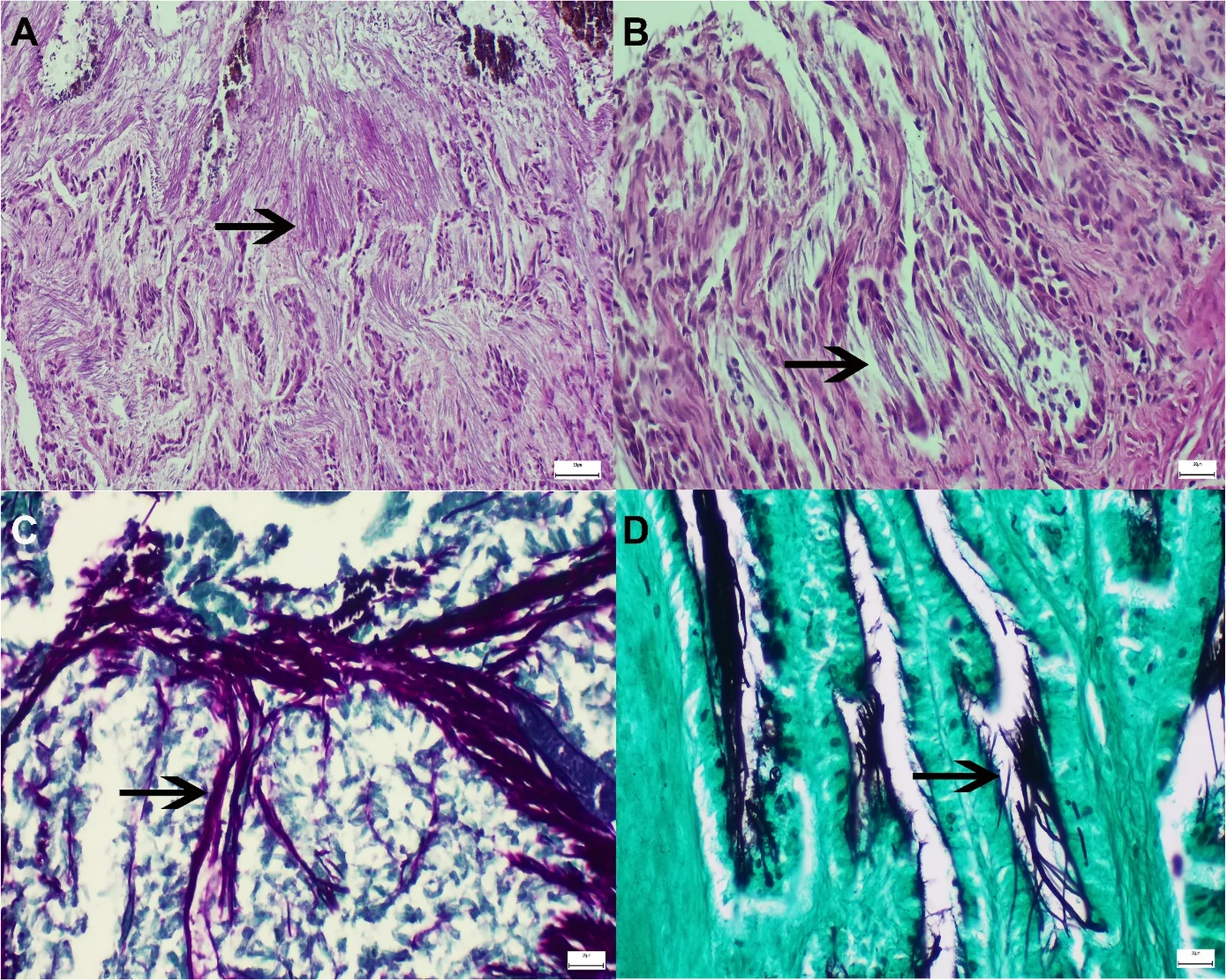
(**A**) Appearance of organisms in the proventriculus and small intestines of budgerigars with macrorhabdosis. Appearance of organisms (arrow) in the proventriculus of a budgerigar, H&E, Bar = 50 μm. (**B**) Close-up view of the organisms (arrow), H&E, Bar = 20 μm. (**C**) Appearance of PAS-stained and (**B**) GMS-stained sections from proventriculus samples, abundant *Macrorhabdus ornithogaster* organisms (arrow) in the proventriculus of a budgerigar, Bars = 20 μm

Histological examination revealed that the most prominent lesions were localized to the proventriculus and, to a lesser extent, the ventriculus. In the majority of examined sections, *Macrorhabdus ornithogaster* organisms were observed along the luminal surface of the proventricular mucosa and within the epithelial crypts. In some cases, organisms were also detected within deeper glandular structures. The distribution and density of *M. ornithogaster* varied among individual birds. While some sections contained only a limited number of organisms, others showed a high concentration within the proventricular mucosa. In sections with increased numbers of organisms, the mucosa frequently exhibited erosive or ulcerative changes, including epithelial discontinuity, mucosal thinning, and focal exposure of the lamina propria (Fig. 2A and B). Inflammatory changes were observed in multiple cases and ranged from mild to marked. These changes were characterized by multifocal to diffuse infiltration of inflammatory cells, predominantly lymphocytes, plasma cells, and heterophils, within the lamina propria and submucosa. The presence and intensity of inflammatory cell infiltration varied among samples. Overall, histopathological alterations were most consistently identified in the proventriculus, whereas lesions in the ventriculus and intestinal segments were less frequently observed.

Histochemical staining allowed visualization of *Macrorhabdus ornithogaster* organisms within proventricular tissues. Periodic acid–Schiff (PAS) staining demonstrated elongated organisms with red to magenta coloration, located on the mucosal surface and within epithelial crypts. The organisms appeared as long, thin, rod-shaped or filamentous structures with relatively uniform staining intensity (Fig. 2C). Gomori methenamine silver (GMS) staining revealed similar organisms as black, slender rods within the proventricular mucosa. Fungal elements were clearly delineated against the surrounding tissue, facilitating their localization in areas with varying degrees of mucosal alteration (Fig. 2D).

**Fig. 3:**
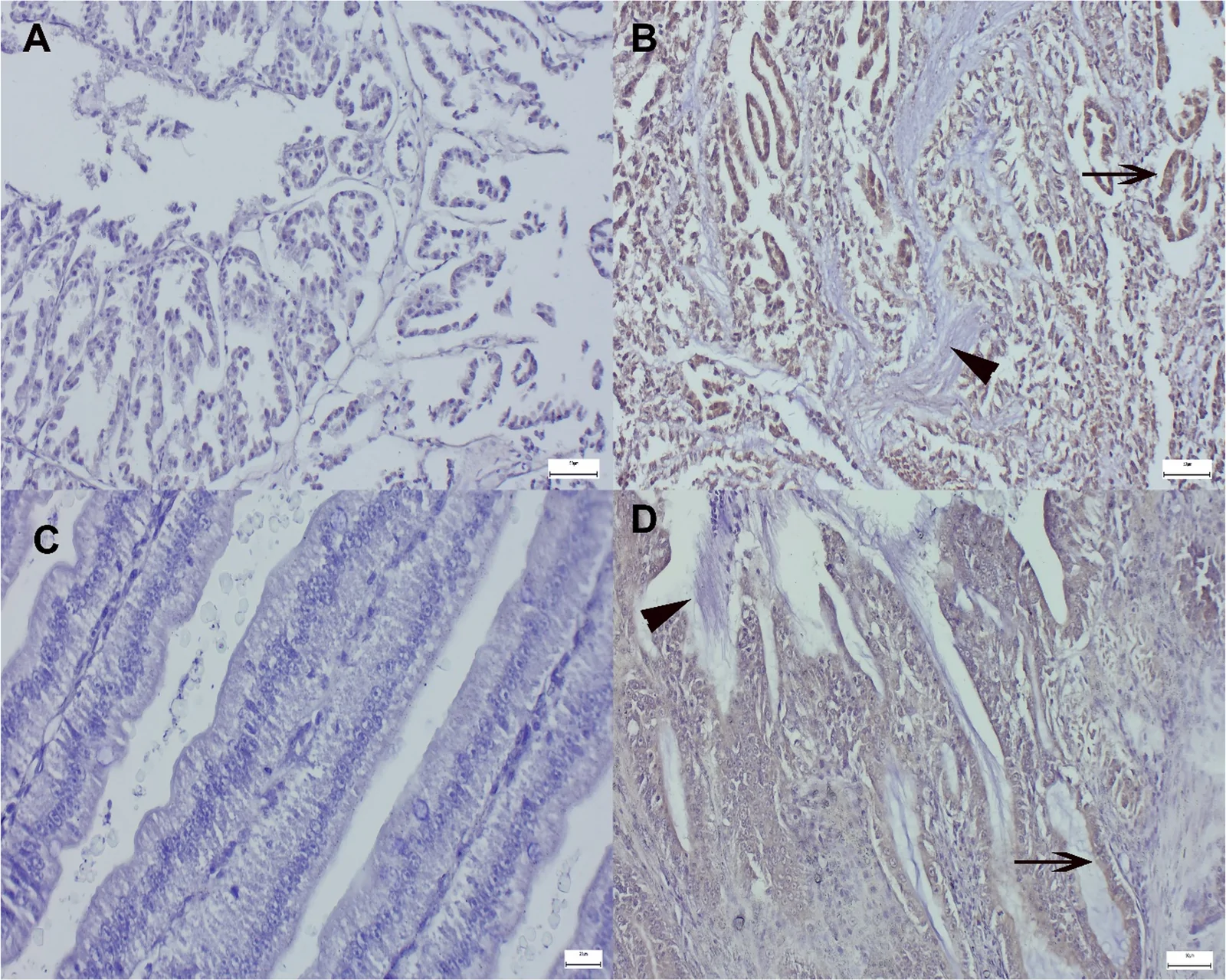
Immunohistochemical localization of TNF-α expression in proventricular and intestinal tissues. (**A**) Absence of TNF-α immunoreactivity in the proventriculus of control birds. (**B**) Marked TNF-α expression (arrow) surrounding *Macrorhabdus ornithogaster* organisms (arrowhead) in infected budgerigars. (**C**) No detectable TNF-α immunostaining in intestinal tissues of the control group. (**D**) Intense cytoplasmic TNF-α immunoreactivity (arrow) observed in epithelial and inflammatory cells in intestinal tissues harboring *M. ornithogaster* organisms (arrowheads). Immunohistochemical staining was performed using the streptavidin-biotin peroxidase method. Bar = 20 μm

Immunohistochemical staining demonstrated TNF-α immunoreactivity in gastrointestinal tissues from birds infected with *Macrorhabdus ornithogaster*. In control tissues and in sections in which fungal organisms were not observed, TNF-α staining was absent or limited to weak, diffuse cytoplasmic labeling. In tissues containing *M. ornithogaster*, TNF-α positive immunoreactivity was detected in multiple cell populations. Positive staining was observed in epithelial cells lining the mucosal surface and glandular structures, as well as in cells within the lamina propria. In areas exhibiting mucosal erosion or ulceration, TNF-α immunoreactivity was also noted in infiltrating inflammatory cells, including macrophage- and lymphocyte-like cells (Fig. 3).

Both PAS and GMS staining consistently demonstrated the presence of *M. ornithogaster* organisms in proventricular sections from affected birds. The distribution and intensity of TNF-α immunostaining varied among tissue sections. TNF-α-positive cells were primarily localized to regions exhibiting histological alterations, whereas adjacent areas without detectable organisms showed minimal or no staining. Negative control sections processed without the primary antibody showed no specific immunoreactivity.

## Discussion

Macrorhabdosis remains a clinically important disease in budgerigars and other avian species, frequently associated with chronic gastrointestinal pathology and poor clinical outcomes. In the present study, macrorhabdosis was evaluated using gross pathological, histological, and immunohistochemical approaches, with particular attention to TNF-α expression in naturally infected birds.

Clinically and macroscopically, affected birds showed emaciation and gastrointestinal lesions predominantly involving the proventriculus, consistent with previous reports (Martins et al. 2006; Baker 1985). Common findings such as mucosal thickening, ulceration, hemorrhage, and excessive mucus production were in agreement with earlier descriptions, supporting disruption of the gastric environment as a key factor facilitating fungal colonization (Gerlach 2001).

Histopathologically, lesions were mainly localized to the proventriculus, with variable involvement of the ventriculus and rare intestinal changes. The absence of neoplastic lesions contrasts with isolated reports of proventricular adenocarcinoma associated with *M. ornithogaster* infection (Phalen 2014; Snyder and Treuting 2014; Powers et al. 2019; Blagojević et al. 2024), suggesting heterogeneity in disease expression possibly related to host factors, infection duration, or strain variability.

Identification of *M. ornithogaster* was supported by H&E, PAS, and GMS staining, which demonstrated characteristic fungal organisms within the proventricular mucosa, in accordance with previous studies (Tomaszewski et al. 2003; Phalen 2005; Kheirandish and Salehi 2011). However, fungal burden was not quantified, and no statistical correlation with lesion severity was performed.

Immunohistochemical analysis demonstrated TNF-α immunoreactivity in epithelial and inflammatory cells in infected tissues, whereas control tissues showed minimal or no staining. Although TNF-α is a key inflammatory mediator in mammals (Beyaert and Fiers 1994; Beutler and Bazzoni 1998), its role in avian fungal infections remains insufficiently characterized. The present findings demonstrate spatially localized TNF-α immunoreactivity within inflamed gastrointestinal tissues colonized by *M. ornithogaster*, supporting the presence of an active mucosal inflammatory response associated with infection.

Several limitations should be acknowledged. The study relied on archived samples from naturally infected birds, resulting in variability in disease stage and tissue availability. Molecular confirmation of *M. ornithogaster* was not performed, and immunohistochemical findings were not supported by quantitative cytokine analyses or statistical evaluation. Another limitation of the present study is the absence of quantitative immunohistochemical scoring or molecular cytokine profiling, which would be valuable for correlating inflammatory mediator expression with lesion severity and fungal burden. Furthermore, only TNF-α was evaluated, while other inflammatory mediators may also contribute to disease pathogenesis.

## Conclusion

The present study demonstrates localized TNF-α immunoreactivity in gastrointestinal tissues of budgerigars naturally infected with *Macrorhabdus ornithogaster*, particularly in areas exhibiting epithelial injury and inflammatory cell infiltration. These findings support the presence of an active local inflammatory response associated with macrorhabdosis and provide additional insight into the pathological alterations accompanying infection. Although the descriptive nature of this study precludes conclusions regarding mechanistic pathways or causality, the observed TNF-α expression highlights the potential involvement of proinflammatory cytokine signaling in host responses to *M. ornithogaster*. Further studies incorporating quantitative immunological and molecular approaches will be necessary to clarify the functional role of cytokines in disease progression and tissue injury in avian macrorhabdosis.

## Data Availability

No datasets were generated or analysed during the current study
